# Methylglyoxal and Advanced Glycation End products: Insight of the regulatory machinery affecting the myogenic program and of its modulation by natural compounds

**DOI:** 10.1038/s41598-017-06067-5

**Published:** 2017-07-19

**Authors:** Mohammad Hassan Baig, Arif Tasleem Jan, Gulam Rabbani, Khurshid Ahmad, Jalaluddin M. Ashraf, Taeyeon Kim, Han Sol Min, Yong Ho Lee, Won-Kyung Cho, Jin Yeul Ma, Eun Ju Lee, Inho Choi

**Affiliations:** 1 Department of Medical Biotechnology, Yeungnam University, Gyeongsan, 38541 Republic of Korea; 2 Faculty of Applied Medical Sciences, Jazan University, Jazan, Saudi Arabia; 3 Department of Biomedical Science, Catholic University of Daegu, Gyeongsan, 38430 Republic of Korea; 4 Korean Medicine (KM) Application Center, Korea Institute of Oriental Medicine (KIOM), Dong-gu, Daegu, 41062 Republic of Korea

## Abstract

Methylglyoxal (MG) is a reactive dicarbonyl intermediate and a precursor of advanced glycation end products (AGEs). The authors investigated the role played by AGEs in muscle myopathy and the amelioration of its effects by curcumin and gingerol. In addition to producing phenotypical changes, MG increased oxidative stress and reduced myotube formation in C2C12 cells. RAGE (receptor for AGEs) expression was up-regulated and MYOD and myogenin (MYOG) expressions were concomitantly down-regulated in MG-treated cells. Interestingly, AGE levels were higher in plasma (~32 fold) and muscle (~26 fold) of diabetic mice than in control mice. RAGE knock-down (RAGE_kd_) reduced the expressions of MYOD and MYOG and myotube formation in C2C12 cells. *In silico* studies of interactions between curcumin or gingerol and myostatin (MSTN; an inhibitor of myogenesis) and their observed affinities for activin receptor type IIB (ACVRIIB) suggested curcumin and gingerol reduce the interaction between MSTN and ACVRIIB. The findings of this study suggest enhanced AGE production and subsequent RAGE-AGE interaction obstruct the muscle development program, and that curcumin and gingerol attenuate the effect of AGEs on myoblasts.

## Introduction

Skeletal muscle is a contractile tissue that consists of cylindrical multinucleated myofibers and resident stem cells, that is, muscle satellite cells (MSCs)^1–3^. Though quiescent, these cells have a remarkable capacity to regenerate muscle and replenish the reserve cell pool. The progression of MSCs along the myogenic lineage is regulated by the co-expressions of paired box transcription factors (Pax3 and Pax7) and the basic helix loop helix transcription factors, MYOD and myogenin (MYOG)^4, 5^, and by regulating the number of cells capable of undergoing proliferation, MSCs regulate myofiber growth. Muscle fiber numbers, which are attributed to the proliferative activity of MSCs during prenatal growth, markedly determine the growth and developmental capacity of postnatal muscles, which have the potential to form multinucleated myotubes and subsequently syncytial contractile myofibers, and thus, MSCs contribute to the maintenance of muscle myofiber size during development^6–8^.

Elevated glucose uptake by skeletal muscles is positively correlated with glucose elimination by the body. However, hyperglycemia leads to the glycation of proteins and enzymes involved in varied cellular processes^9, 10^. Furthermore, the endogenous metabolites, methylglyoxal (MG), glyoxal, and 3-deoxyglucosone (3-DG), which are generated by the auto-oxidation of glucose, are reactive glycating agents and reduce sugars to produce advanced glycation end products (AGEs)^11–13^. Non-enzymatic glycation starts with the formation of Schiff bases, which are converted into amadori products and then to AGEs^14^, and the progressive accumulation of AGEs in tissues and organs induces the produce reactive oxygen species (ROS) and causes oxidative stress, which mainly arises from mitochondrial dysfunction^15, 16^. AGE-modified proteins may exert their effects by binding to RAGE (AGE-specific cell surface receptors), and elevated RAGE expression has been reported on the endothelial cells, vascular smooth muscle cells, and cardiac myocytes of diabetic patients^17^. Furthermore, interactions between AGEs and RAGE have been reported to activate intracellular signaling, induce gene expression, produce pro-inflammatory cytokines and free radicals, and thus, this interaction has been suggested to play an important role in the development and progression of diabetes^18^.

Diabetic myopathy is characterized by low muscle mass, weakness, and reduced physical capacity, and is increasingly becoming a leading cause of morbidity and mortality^19, 20^. Patients with diabetic myopathy usually have other long-term manifestations of diabetes, such as, peripheral vascular disease, nephropathy, and cardiovascular diseases^21–23^. Nutritional interventions in patients with diabetes have been reported to induce significant improvement in a series of functional and physiological parameters^24, 25^. In particular, dietary supplementation of natural compounds, especially curcumin and gingerol, has been reported to ameliorate the complications associated with diabetes^26, 27^. Up-holding strong antioxidant activity, they extend beneficial effects in reducing the occurrence of diabetes, cardio-metabolic health, inflammatory response and neurodegenerative diseases^24, 27–29^.

Increased MG production arising from hyperglycemia has been reported to be responsible for the carbonyl stress associated with vascular damage in diabetes^17, 30^. However, despite numerous studies on the topic, little information is available about the stages of the myogenic process affected by AGEs. Accordingly, the present study was undertaken to explore the antagonistic roles of curcumin and gingerol on the effect of MG. An *in silico* study of curcumin and gingerol showed higher gold fitness score for possible interaction with the negative regulator, myostatin (MSTN). The binding of MSTN to activin receptor type IIB (ACVRIIB) helps to keep the myogenic program in check^3^. In the present study, we sought to elucidate the mechanism responsible for AGE mediated decrease in muscle mass, to determine the effects of curcumin and gingerol supplementation on AGE production, and relationships between increased AGE production, functional impairments of myoblasts, and their possible reversal by curcumin or gingerol in diet.

## Results

### Estimation of AGEs in MG treated cells

**Figure 1:**
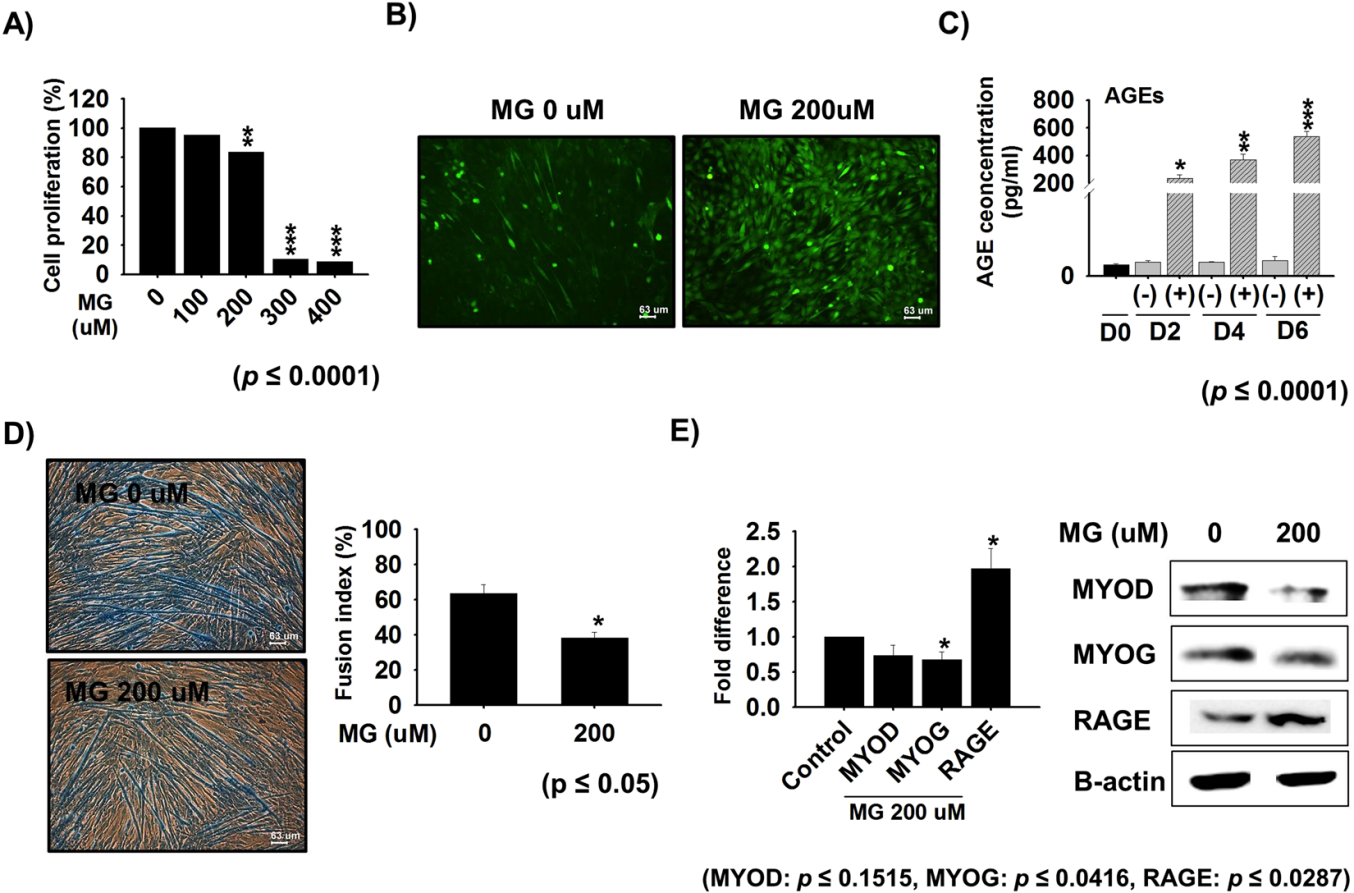
Association between MG induced AGE production and myogenesis. (**A**) Cell proliferation in the presence of different concentrations of MG at Day 4. (**B**) Cells were cultured with or without 200 µM MG for 4 days in differentiation media and ROS levels were assessed using DCFDA by florescence microscopy (n = 3) (**C**) AGE levels after treatment with 200 µM MG in differentiation media for 0, 2, 4, or 6 days [(−): MG non-treated cells, (+): MG treated cells] (**D**) Myotube formation and fusion indices of cells treated with or without 200 µM MG for 4 days in differentiation media (**E**) MYOD, MYOG and RAGE expressions determined by real time PCR and Western blot (n = 3).

In a preliminary study, we used the MTT assay to investigate the cytotoxicity of MG. C2C12 cells (35–40% confluent) were treated with different concentrations of MG (100, 200, 300 or 400 µM) and assayed after 4 days of treatment. MG at 200 µM was found to be non-toxic (Fig. 1A﻿, supplementary Fig S1), and thus this concentration was selected to access the effects of MG on myogenesis. The oxidative stress in MG-treated and non-treated control cells cultured in differentiation medium for 4 days were assessed by measuring DCFDA fluorescence intensities, and oxidative stress levels were found higher in MG (200 µM) treated cells than in the controls **(**Fig. 1B **)**. C2C12 cells treated with 200 µM MG for 0, 2, 4 or 6 days showed gradual increases in AGE levels by ELISA, whereas AGE levels remained unchanged in controls **(**Fig. 1C **)**.

### Effect of MG on cellular differentiation

Treatment of C2C12 cells with MG (200 µM) for 4 days reduced fusion indices by 55–60% compared with non-treated controls **(**Fig. 1D **)**, and induced morphological changes and reduced myotube formation. Furthermore, MYOD and MYOG expressions were significantly decreased at the mRNA and protein levels in MG treated cells (Fig. 1E).

### AGE levels in mouse tissues

**Figure 2:**
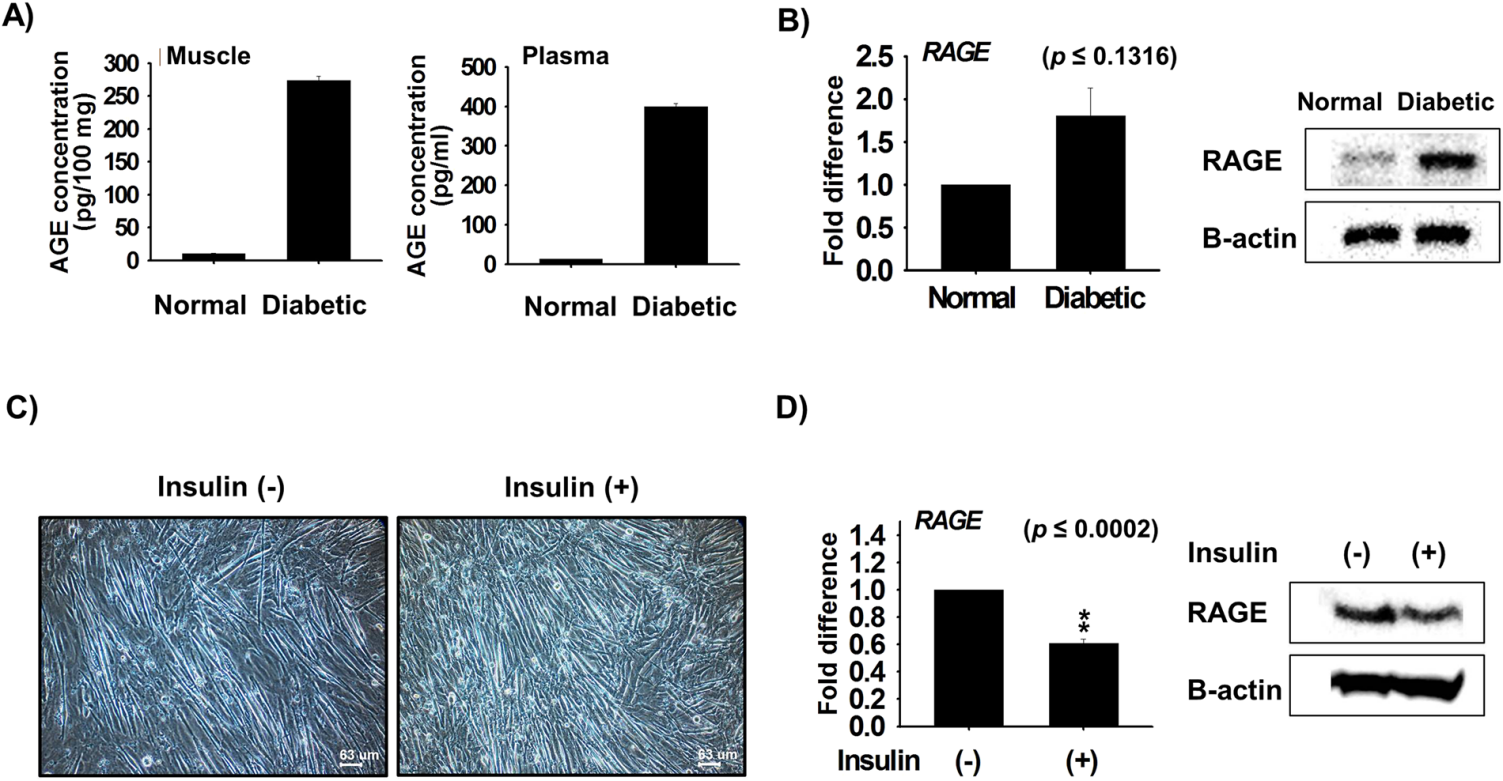
AGE levels in diabetic and non-diabetic C57BL/6 mice. Levels of AGEs in diabetic and non-diabetic mice (**A**) Muscle and Plasma (n = 3, P < 0.05). (**B**) RAGE expression as analyzed by real time PCR and Western blot in cells treated with 200 µM MG for 4 days in differentiation media. (**C** and **D**) Effect of insulin supplementation on cell differentiation and RAGE expression as determined by real time PCR and Western blot (n = 3).

AGE concentrations were estimated in diabetic mouse muscle and plasma by ELISA. Considerable amounts of AGEs were detected in plasma (399 ± 9.01 pg/ml plasma) and muscle (274 ± 6.65 pg/100 mg tissue), whereas amounts were negligible in the non-diabetic mice **(**Fig. 2A **)**.

### RAGE protein and mRNA expressions and RAGE knock-down

To determine effect of MG on RAGE expression during differentiation, C2C12 cells were cultured in differentiation media for 4 days. We examined the effect of insulin supplementation on cell differentiation. We found numbers of myotubes were higher in insulin supplemented cells than in non-treated cells (Fig. 2C). Furthermore, RAGE expression was reduced in insulin supplemented cells at the mRNA and protein levels (Fig. 2D). In addition, a separate analysis revealed the mRNA and protein levels of RAGE were higher in diabetic mouse tissues than in non-diabetic controls (Fig. 2B).

**Figure 3:**
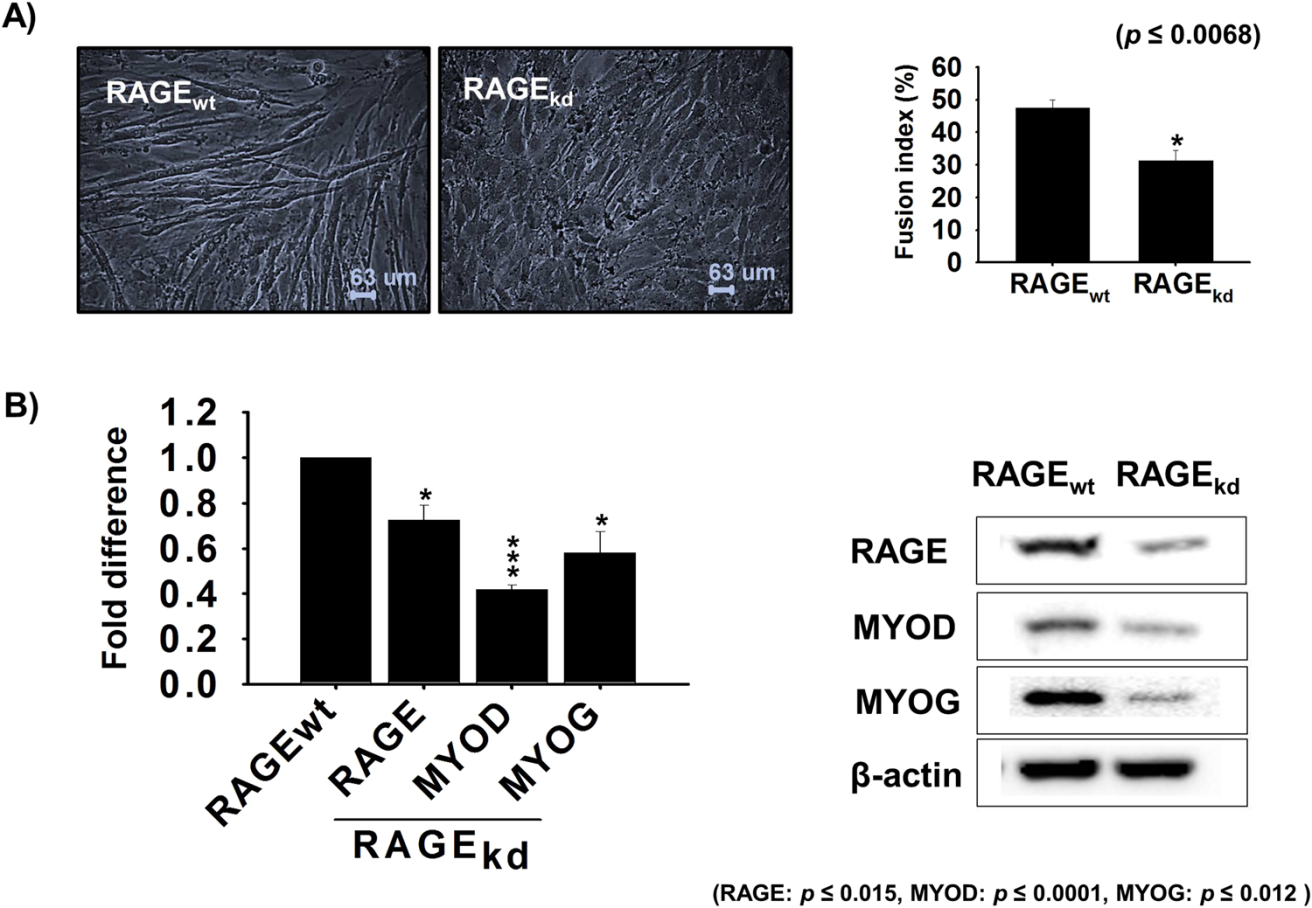
RAGE gene expression and the effects of its knock-down during differentiation. RAGE knock-down in C2C12 cells (**A**) Effect of RAGE knock-down on differentiation as determined by myotube formation and by using fusion indices. (**B**) RAGE, MYOD, and MYOG expressions in RAGE_kd_ cells were determined by real time PCR and Western blot (n = 3).

To access the function of RAGE during myogenesis, we used a RAGE knock-down approach in C2C12 cells. Myotube formation by RAGE knock-down (RAGE_kd_) cells cultured in differentiation media for 4 days was less than demonstrated by RAGE_wt_ (scrambled vector) transfected cells (Fig. 3A). Comparison of fusion indices of RAGE_kd_ and RAGE_wt_ cells gives indication of the effect of RAGE_kd_ on myotube formation. Furthermore, the protein and mRNA expressions of RAGE, MYOD, and MYOG were lower in RAGE_kd_ cells than in RAGE_wt_ cells (Fig. 3B). These results suggest increases in AGEs due to reduced RAGE expression adversely affect myotube formation and the myogenic developmental program.

### Effects of curcumin and gingerol on myogenesis

**Figure 4:**
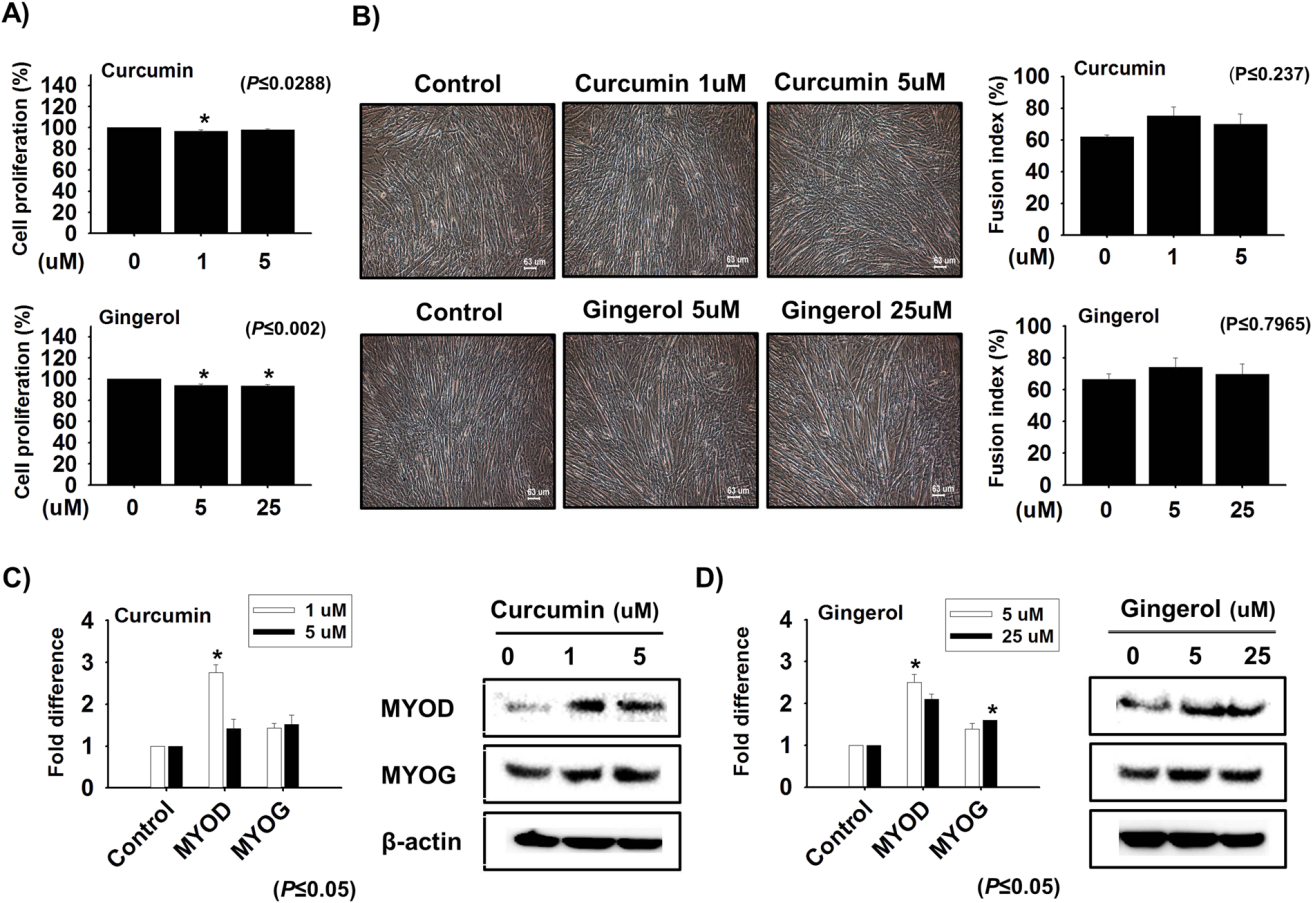
Effect of curcumin and gingerol on cellular differentiation. (**A**) Cell proliferations in the presence of different concentrations of curcumin (1 or 5 µM) or gingerol (5 or 25 µM). (**B**) Effects of curcumin or gingerol on myotube formation and fusion indices. (**C** and **D**) MYOD and MYOG expression determination by real time PCR and Western blot after treating cells with different concentrations of curcumin or gingerol (n = 3).

The effects of different concentrations of curcumin and gingerol on the proliferation and differentiation C2C12 cells were examined. At curcumin and gingerol concentrations of 1 μM and 5 μM, respectively, both compounds were non-cytotoxic (Fig. 4A). At these concentrations, curcumin and gingerol both promoted differentiation as evidenced by fusion indices (Fig. 4B, supplementary Fig S2). The mRNA expression of MYOD was increased by 1 μM curcumin or 5 μM gingerol (Fig. 4C and D). Compared with increase in MYOG expression at 25 μM gingerol, 5 μM curcumin was found to exert less effect on the expression of MYOG (Fig. 4C and D). These findings show curcumin and gingerol both promoted myoblast differentiation but not proliferation.

### *In silico* studies of interactions between curcumin and gingerol and myostatin

**Figure 5:**
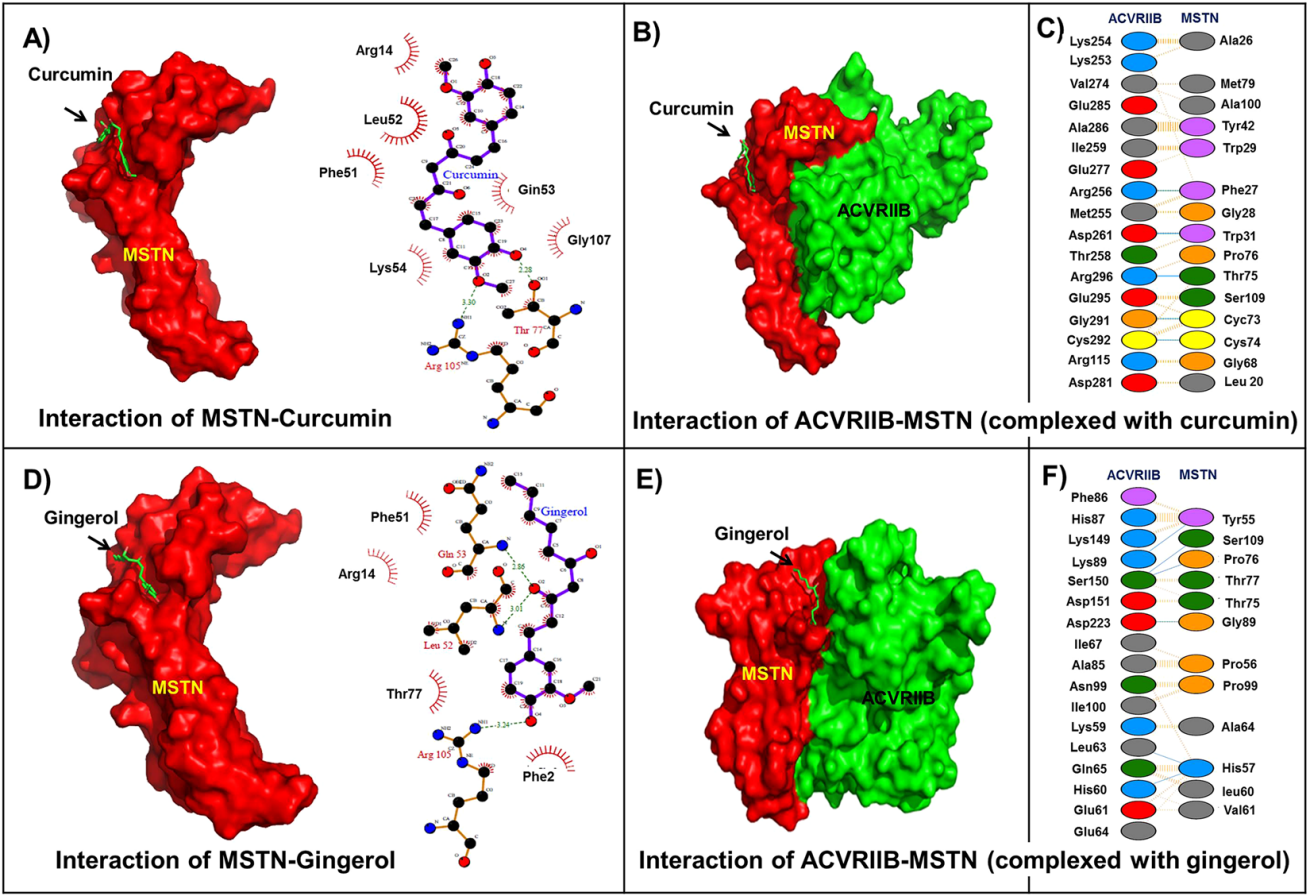
Interactions between curcumin or gingerol with myostatin and their effects on ACVRIIB. (**A**) Residual interaction between curcumin and MSTN in MSTN-curcumin complex (**B**) Interaction between ACVRIIB and MSTN-curcumin complex (**C**) Residual interaction between ACVRIIB and MSTN-curcumin complex (**D**) Residual interaction between gingerol and MSTN in MSTN-gingerol complex (**E**) Interaction between ACVRIIB and MSTN-gingerol complex (**F**) Residual interaction between ACVRIIB and MSTN-gingerol complex.

To investigate the involvement of curcumin and gingerol in the myogenic program protein-ligand and protein-protein interactions studies were performed to access regulation of the interaction between MSTN and ACVRIIB. A series of natural compounds were in silico screened against MSTN using GOLD. On the basis of their goldfitness score, curcumin and gingerol were found to be the the best compounds (Table 1). To gain an insight of the mechanism involved, MSTN-curcumin (Fig. 5A) and MSTN-gingerol complexes (Fig. 5D) were docked to ACVRIIB (Fig. 5B and E). In the absence of curcumin and gingerol, MSTN interacted with ACVRIIB with a global free energy score of −56.99 ^3^. However, a decrease in the global free energy of MSTN to ACVRIIB binding to −46.55 and −47.26 was observed for MSTN-curcumin and MSTN-gingerol complexes, respectively, which suggest curcumin and gingerol interfere with interaction between MSTN and ACVRIIB (Fig. 5C and F). Table 2 provides information about amino acid residues involved in the binding of MSTN (complexed with curcumin or gingerol) to ACVRIIB.

**Table 1:** Binding scores of selected natural compounds and the residues involved in their interaction with MSTN.

Compound	Gold fitness score	X-score	Residues involved	
			Hydrogen bond	Hydrophobic interaction
Curcumin	40.15	−6.86	T77, R105	R14, F51, L52, Q53, K54, T77, R105, G107
Gingerol	40.48	−6.47	L52, QQ53, R105	F22, R14, F51, L52, Q53, T77, R105

**Table 2:** Residues involved in the binding of MSTN (in the presence of curcumin and gingerol) to its receptor ACVRIIB.

Complex	Residues involved			
	Hydrogen bonding		Hydrophobic interaction	
	ACVRIIB	MSTN	ACVRIIB	MSTN
ACVRIIB-MSTN (Curcumin)	R256, D261, G291, C292, R296	F27, W31, C73, C74, T75	R115, S118, K253, K254, M255, R256, T258, I259, D261, V274, E277, W280, D281, D283, E285, A286, G291, C292, E294, E295, R296	L20, A26, F27, G28, W29, W31, Y42, G68, A70, C73, C74, T75, P76, Y86, F87, A100, S109
ACVRIIB-MSTN (Gingerol)	H60, L63, K89, S150, D223	H57, Y55, P76, S109, G89	D31, F32, K59, H60, E61, L63, L64, Q65, G66, I67, T84, A85, F86, H87, D88, K89, N99, I100, L148, K149, S150, D151, D223	Q53, K54, Y55, P56, H57, L60, V61, A64, P76, T77, M79, G89, P99, S109

## Discussion

AGEs produced by the glycation of proteins act as link between hyperglycemia and the development of diabetes. AGEs levels have been reported to influence morbidity rates associated with the complications of diabetes, such as, nephropathy, neuropathy, and cardiovascular diseases^19^. Although the etiologies of type 1 and type 2 diabetes mellitus are multifaceted, AGEs are considered to be a major factor of the development of secondary complications^31^. Increased MG levels, which result in the tissue accumulation of AGEs, have been implicated in the initiation of various pathophysiological disorders^32^. Intracellular accumulation of MG and the subsequent glycation of biomolecules results in oxidative stress, chronic low-grade inflammation, and impaired extracellular matrix (ECM) remodeling^23^. In diabetic mice, elevated AGE levels have been suggested to be associated with disease severity. To investigate the effect of AGEs on muscle development, we treated C2C12 cells with different concentrations of MG. We found MG treatment enhanced AGE generation interfered with myoblast dynamics by impeding their ability to proliferate and differentiate, and thus, reduces muscle recovery.

Protein glycation, which leads to AGE production, is considered a major cause of the complications of diabetes, and MG-induced AGE production accelerates the accumulations of pathogenic species that play critical roles in the development of diabetic myopathy^33^. In the present study, MG treatment induce production of AGEs in C2C12 cells, and ROS levels were higher in MG-treated cells than in non-treated controls, and we found AGE levels were elevated in the plasma and muscle of diabetic mice. Compared to control, increase in RAGE was observed under both *in vitro* and *in vivo* conditions. Some authors have suggested interactions between AGEs and RAGE lead to the activation of NF-kB, elevate oxidative stress, and increase the levels of inflammatory factors^34^. Furthermore, available evidence suggests that increased ROS production resulting from AGE-RAGE interactions is due in part to the activation of NADPH oxidase^35–37^. Several other *in vitro* studies have also shown AGE-RAGE interactions modulate cellular properties^38, 39^. In the present study, oxidative stress resulting from AGE-RAGE interaction appeared to be primarily responsible for the inhibition of myotube formation by causing reduction in the expression of myogenic markers.

To further understand the role played by RAGE in muscle development, we examined the effects of AGEs on cellular differentiation by investigating myotube formation by RAGE_wt_ and RAGE_kd_ C2C12 cells. Observed morphological changes in MG treated cells, such as, reduced fusion rates and increased cell death, showed MG induced AGE production adversely affected myotube formation. Furthermore, the reduction in MYOD expression in MG treated cells also indicated AGEs had an inhibitory effect on myotube formation. Our findings indicate RAGE importantly controls cellular events in the myogenic program, and provide first evidence that the inhibitory effect of MG on myogenesis involves the up-regulation of AGE production. Several studies have reported that curcumin and gingerol beneficially inhibit AGE increases^40^. Curcumin and gingerol have been reported to attenuate protein degradation through the ubiquitin-proteosome pathway via NF-kB and to enhance antioxidant activity by increasing serum glutathione peroxidase (GPx), sulfoxide dismutase (SOD), and glutathione (GSH) levels^41, 42^.

As curcumin and gingerol exhibit a wide variety of beneficial effects, it seems imperative to screen them for their effects on muscle development. In the present study, an investigation of their effects on C2C12 cells indicated they regulate MYOD, MYOG and RAGE genes, which agrees well with the findings of *in vivo* study, in which curcumin or gingerol administration increased muscle regeneration and reduced muscle atrophy and increases glucose metabolism to counteract the effect of high fat diet induced obesity in mouse models^24, 40, 43–45^. *In silico* binding study was performed to investigate the nature of interactions between curcumin, gingerol and MSTN. A large number of plausible binding modes were detected and ranked on the basis of their gold fitness scores, and were re-scored to confirm the accuracy of binding using X-score determined energies. The gold fitness score of curcumin and gingerol were 40.15 and 40.48, while their X-score was found to be −6.86 and −6.47 kcal/mol, respectively. (Table 1) Interaction study between MSTN and ACVRIIB also indicated that curcumin or gingerol interfere with MSTN to ACVRIIB binding.

**Figure 6:**
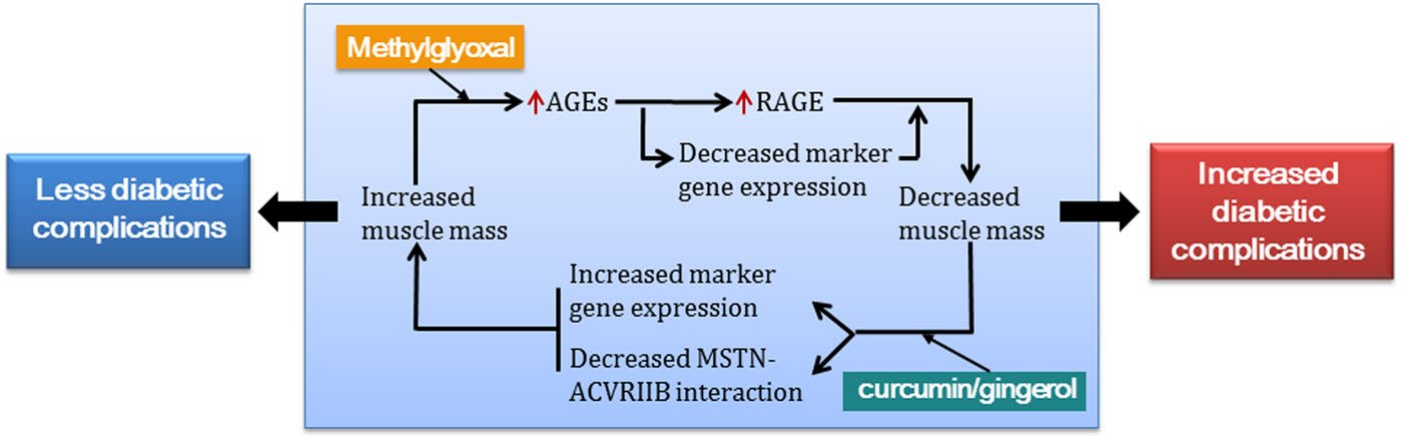
Hypothetical depiction of the adverse effects of AGEs on muscle in diabetes and the inhibition of these effects by dietary curcumin or gingerol supplementation.

Our observation that AGE enhanced ROS production supports the notion that AGE regulates myogenic differentiation in a diabetic background (Fig. 6). Our results indicate: (1) MG-mediated induction of AGEs leads to oxidative stress in myoblasts; (2) Increased AGE levels significantly reduce myotube formation and expressions of myogenic marker genes; (3) AGE levels are positively associated with RAGE expression and negatively associated with muscle mass; (6) Curcumin and gingerol both augment the cellular antioxidant pool and thereby help inhibit AGE production; and that (7) Curcumin or gingerol binding to MSTN inhibits binding with ACVRIIB.

## Material and Methods

### Reagents, laboratory animals, and the induction of diabetes

MG, curcumin, and gingerol were purchased from Sigma Aldrich (St Louis, MO, USA). Stock solutions of MG, curcumin, and gingerol were prepared in dimethyl sulfoxide (DMSO, Sigma Aldrich) and stored at 4 °C until used.

C57BL/6 male mice were purchased from Daehan Biolink (Eumseong, Korea) and housed 3–4 per cage in a temperature-controlled room under a 12-hr light/12-hr dark cycle. Animals were allowed free access to a normal diet (ND) containing 4.0% (w/w) total fat (Rodent NIH-31 Open Formula Auto; Zeigler Bros., Inc., Gardners, PA) and to water. All animal experiments were performed after receiving protocol approval from the Institutional Animal Care and Use Committee (IACUC) of the Catholic University of Daegu (CUD IACUC-2014-035). In addition, this protocol complied with the NIH Guide for ‘*The Care and Use of Laboratory Animals*’. Diabetes was induced using a multiple low-dose streptozotocin (STZ, Sigma-Aldrich, MO, USA) induction protocol as devised by the Animal Models of Diabetic Complications Consortium^46^. Briefly, a solution of STZ (0.75%) in 0.1 M sodium citrate buffer (pH 4.5) was injected intraperitoneally (i.p.) into 21-week-old mice for 5 consecutive days. Non-diabetic and diabetic mice at 26 weeks old were anesthetized using sodium pentobarbital (i.p.), and blood samples were collected by cardiac puncture. Blood samples were centrifuged at 2,400 × g for 15 min at 4 °C, and the plasma obtained was then centrifuged at 12,500 × g for 15 min. In addition, rectus femoris muscles were collected after sacrifice^47^.

### Cell Culture and fusion index

Murine C2C12 myoblast cells were cultured in DMEM (Dulbecco’s Modified Eagle’s Medium; HyClone Laboratories, UT, USA) supplemented with 10% FBS (fetal bovine serum, HyClone Laboratories) and 1% penicillin/streptomycin (P/S; HyClone Laboratories) at 37 °C in a humid 5% CO_2_ atmosphere. To assess the effect of MG (Sigma Aldrich) on myoblast differentiation, cells at 70% confluence were cultured in differentiation media (DMEM + 2%FBS + 1%P/S) with or without MG for 0, 2, 4 or 6 days. In addition, the effects of curcumin (1 or 5 μM) or gingerol (5 or 25 μM) on myogenesis were examined in C2C12 cells. For insulin effects, cells at 100% confluence were cultured for 2 days in DMEM +1%P/S with or without insulin. Media were changed every other day.

Fusion indices were calculated as previously described by Brigitle *et al*.^48^. Briefly, cell nuclei were stained with Giemsa G250 (Sigma Aldrich) and pictures were captured randomly at three different locations. Numbers of nuclei in myotubes and total numbers of nuclei in cells were counted in each field. Fusion indices were calculated by expressing total numbers of nuclei in myotubes as percentages of total numbers of nuclei.

### RNA extraction and real time PCR analysis

Total RNA was extracted as previously described using Trizol^™^ reagent (Invitrogen, CA, USA)^2^. cDNA was synthesized using a High capacity cDNA reverse transcription kit (Applied Biosystems, CA, USA). Briefly, 2 µg of RNA in a 20 µl reaction mixture was primed with random hexamer and reverse transcribed in a thermocycler programmed at 25 °C for 10 min, 37 °C for 120 min, and 85 °C for 5 min. Real time PCR was performed using 2 µl of cDNA, 10 pmol of each gene-specific primer and Power SYBR^®^ Green PCR MasterMix (Applied Biosystems) on a 7500 real-time PCR system (Applied Biosystems). Primers employed in this study were designed using Primer 3 software (http://frodo.wi.mit.edu) using sequence information listed at the National Center for Biotechnology Information. Primer sequence information is provided in Supplementary Table S1.

### Western blot analysis

Western blot was performed as described by Lee *et al*.^49^. Briefly, cells were washed with ice cold PBS and lysed using RIPA buffer containing protease inhibitor cocktail (Thermo Scientific, NH, USA). Total proteins were isolated by centrifugation at 13,000 rpm for 10 min at 4 °C and quantified using the Bradford method. Briefly, proteins (40 µg) were heated at 90 °C for 5 min with ß-mercaptoethanol (Sigma-Aldrich), electrophoresed in 10% SDS-polyacrylamide gels, and transferred to PVDF membranes. Blots were blocked with 3% skimmed milk in TBST (Tris-Buffered Saline and Tween 20) for 1 hr, incubated overnight with either MYOD (1:400), MYOG (1:400), RAGE (1:400), or β-actin (1:1000) antibody (Santa Cruz Biotechnology), diluted with 1% skimmed milk in TBS (Tris-Buffered Saline) at 4 °C, washed with TBST, and incubated with horseradish peroxidase conjugated secondary antibody (Goat anti rabbit or mouse antibody, Santa Cruz Biotechnology) for one hour at room temperature. After a final wash with TBST, blots were developed using Super Signal West Pico Chemiluminescent Substrate (Thermo Scientific).

### RAGE knock-down

RAGE gene knock-down was performed in C2C12 cells as described by Kamli *et al*.^50^. Briefly, C2C12 cells grown in 6-well plates to 30% confluence were transfected with 1 ng/well of scrambled shRNA (RAGE_wt_, the control) or a RAGE shRNA construct (RAGE_kd_; Santa Cruz Biotechnology, CA, USA) using transfection reagent and transfection medium (Santa Cruz Biotechnology). After 3 days, cells were selected with 2 μg/ml of Puromyocin (Santa Cruz Biotechnology), and selected cells were grown to 70% confluence then switched to differentiation medium. The sequence of the shRNA construct is provided in Table S2.

### Cell proliferation assay

C2C12 cells were cultured in proliferation medium (DMEM + 10%FBS + 1%P/S) containing 0, 100, 200, 300, or 400 μM of MG for 4 days and then proliferation was determined using a MTT assay. Briefly, cells grown on 6 well plates were treated with different concentrations of MG for 4 days and then incubated for 2 hr with MTT reagent (0.5 mg/ml; Sigma Aldrich). The formazan crystals produced were dissolved in DMSO and absorbance was measured at 540 nm (Tecan Group, Switzerland).

### ELISA

Total AGE levels in C2C12 cells (treated with or without MG for 0, 2, 4, 6 days during myogenesis) and in plasma and muscle tissue were measured using an ELISA kit (NeoBiolab, MA, USA). Briefly, homogenized muscle tissues or plasma or cells with and without MG were added with enzyme assay reagent to specific antibody-coated microtiter plates and then incubated for 30 min at room temperature. Enzyme conjugate was then added and plates were incubated further for 30 min at room temperature. Mixtures were then discarded and plates were washed to remove unbound materials. Substrate solution was then applied and left for 20 min. The reaction was terminated by adding stop solution and color intensities were measured by taking absorbance at 450 nm.

### Assessment of intracellular ROS levels during myogenesis

C2C12 cells cultured for 4 days in differentiation media containing 200 μM MG were assessed for ROS production using DCFH-DA (2ʹ−7ʹ-dichlorodihydrofluorescein diacetate, Sigma Aldrich) as described by Kamli *et al*.^50^. Briefly, after culture media had been removed, cells were washed with DMEM, incubated with 10 μM DCFH-DA in DMEM for 30 min at 37 °C, and then washed with PBS. DCFDA fluorescence intensities were assessed by fluorescence microscopy (Nikon).

### Preparation of template and ligands for docking

The three-dimensional protein structure of MSTN was retrieved from the RCSB protein databank (pdb id: 3HH2)^51^. Due to the non-availability of 3D the structure of ACVRIIB, we generated its 3D-structure using amino acid sequence (Uniprot id: P27040). Modeller 9v14^52^ was employed to generate a structure of ACVRIIB by homology modelling using the crystal structure of human activin receptor type II kinase domain (pdb id: 2QLU) as a template. Discrete optimized protein energy (DOPE) scores were used to select the best five models of ACVRIIB. The 3D structures of about 30 natural compounds with reported activity in muscle formation were extracted from the PubChem compound database.

### Molecular Docking

Molecular docking was used to investigate the interactions between selected natural compounds and MSTN. Genetic Optimization for Ligand Docking 5.0 (GOLD)^53^ was used to dock selected natural compounds. Annealing parameters were set at 5.0 and 2.5 to evaluate van der Waals and hydrogen bonding, respectively. The population size was set at 100 at a selection pressure of 1.2. The number of operations was fixed at 1,00,000, with 5 islands, niche size 2, migration value of 10, mutation value of 100, and a cross-over of 100. The binding energies of docked molecules were calculated using X-score^54^. Finally, curcumin and gingerol were selected for investigations based on their gold fitness scores and X-score values. Molecular graphics of docked complexes were prepared using Pymol.

### Protein-protein interactions

To investigate the binding of ACVRIIB to MSTN (uncomplexed and complexed with selected natural compounds), a protein-protein interaction study was performed using PatchDock server (http://bioinfo3d.cs.tau.ac.il/PatchDock/)^55^. The results obtained from PatchDock were subjected to further refinement using Firedock^56^, and complexes with maximum interaction energies were selected for further studies. Differences between the interaction energies of MSTN (complexed and uncomplexed) with ACVRIIB were used to investigate the effects of curcumin and gingerol on binding affinity between MSTN and its receptor ACVRIIB.

### Statistical Analysis

Means of normalized expressions were compared using Tukey’s Studentized Range (HSD) to determine the significances of gene expression differences. Real time PCR data were normalized using GAPDH as an internal control and analyzed by one-way ANOVA using PROC GLM in SAS ver. 9.0 (SAS Institute, Cary, NC, USA). P-values of <0.05 were considered statistically significant.

## Electronic supplementary material

Supplementary info
