# Supplementary material for: Methylglyoxal and Advanced Glycation End products: Insight of the regulatory machinery affecting the myogenic program and of its modulation by natural compounds
Source: Sci Rep. 2017 Jul 19;7:5916. doi: 10.1038/s41598-017-06067-5 (PMC5517486; doi:10.1038/s41598-017-06067-5)
Supplement: Supplementary file 1 — Supplementary info [file 41598_2017_6067_MOESM1_ESM.pdf]

Methylglyoxal and Advanced Glycation End products: Insight of the regulatory machinery affecting the myogenic program and of its modulation by natural compounds

Mohammad Hassan Baig[1#], Arif Tasleem Jan[1#], Gulam Rabbani[1], Khurshid Ahmad[1], Jalaluddin M Ashraf[1], Taeyeon Kim[1], Han Sol Min[2], Yong Ho Lee[2], Won-Kyung Cho[3], Jin Yeul Ma[3], Eun Ju Lee[1*], Inho Choi[1*]

[1]Department of Medical Biotechnology, Yeungnam University, Gyeongsan, 38541, Republic of Korea

[2]Department of Biomedical Science, Catholic University of Daegu, Gyeongsan, 38430, Republic of Korea

[3]Korean Medicine (KM) Application Center, Korea Institute of Oriental Medicine (KIOM), Dong-gu, Daegu 701-300, Republic of Korea

LEGENDS

Supplementary Table 1: Primer information corresponding to the ORF of different genes.

Supplementary Table 2: shRNA construct information used for RAGE knock-down.

Supplementary Figure 1. Cell morphology and proliferation following treatment of C2C12 cells with different concentrations of MG.

Supplementary Figure 2. MYL2 and MYH4 expression in C2C12 cells following treatment with curcumin or gingerol.

Supplementary Table 1

Species	Gene	Product size (bp)	Tm (°C)	Sequence (F)	Sequence (R)
Mouse	GAPDH	155	55	5'-tgctggtgctgagtatgtcg-3'	5'-caagcagttggtggtacagg-3'
	RAGE	160	59	5’-aggaacgtgcagagctgaat-3’	5’-ctggttggagaaggaagtgc-3’
	MYOD	213	59	5'-aggagcacgcacacttctct-3'	5'-tctcgaaggcctcattcact-3'
	MYOG	185	59	5'-tccagtacattgagcgccta-3'	5'-caaatgatctcctgggttgg-3'
	MYL2	177	59	5'-aaagaggctccaggtccaat-3'	5'-cctctctgcttgtgtggtca-3'
	MYH4	248	59	5'-gggttccattgacattgacc-3'	5'-agggccagtgtttcacattc-3'

Supplementary Table 2

shRNA	Sequence	
RAGEshRNA (SC-36375-SH)	RAGE shRNA Plasmid (m) is a pool of 3 different shRNA plasmids	
	sc-36375-SHA	GATCCGAACACAGGAAGAACTGAATTCAAGAGATTCAGTTCTTCCTGTGTTCTTTTT
	sc-36375-SHB	GATCCGATTCCCGATGGCAAAGAATTCAAGAGATTCTTTGCCATCGGGAATCTTTTT
	sc-36375-SHC	GATCCGCATTCAGCTGTTGGTTGATTCAAGAGATCAACCAACAGCTGAATGCTTTTT

Supplementary Figure 1.

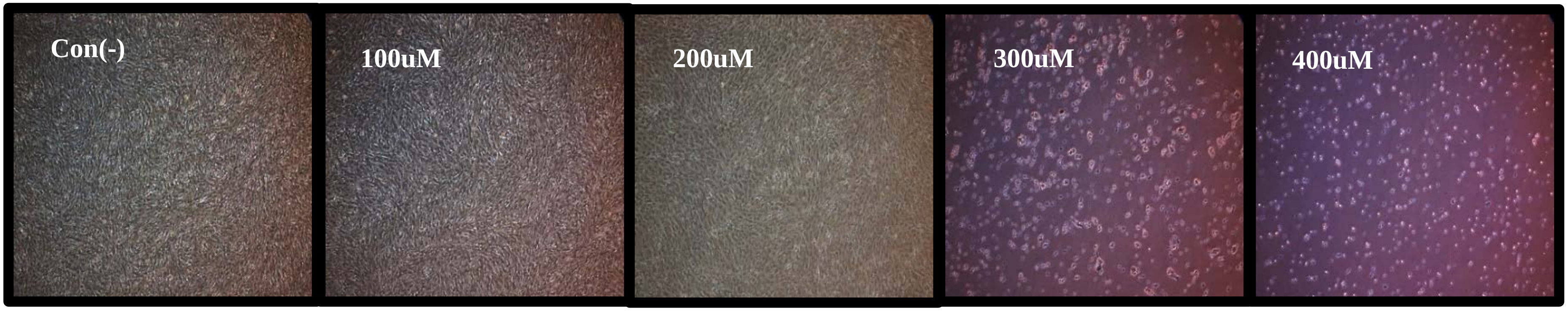

Supplementary Figure 2.

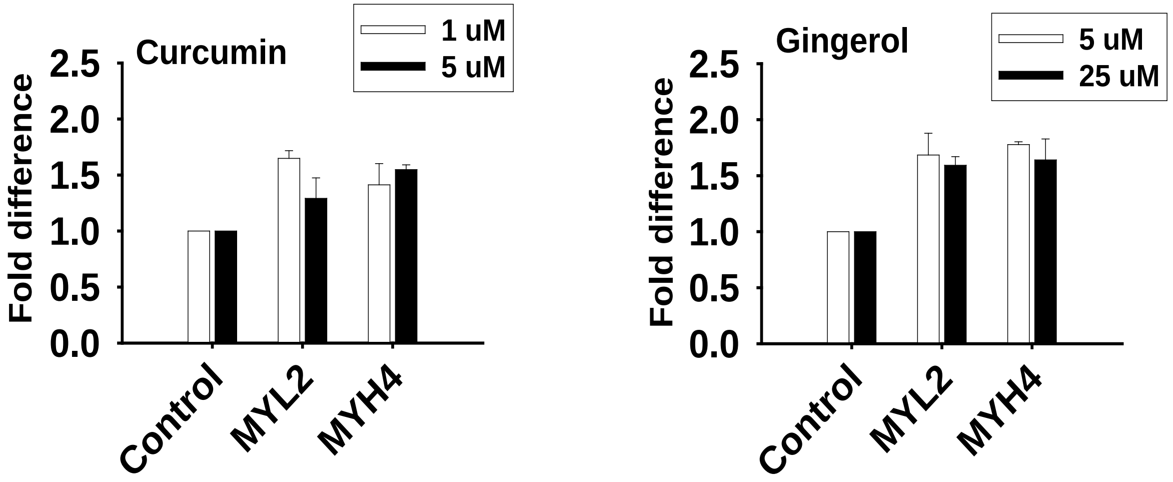

(MYL2: *P*≤0.0579, MYH4: *P*≤0.0812)

(MYL2: *P*≤0.0502, MYH4: *P*≤0.0301)
